# Knowledge Mapping of the Literature on Fiber-Reinforced Geopolymers: A Scientometric Review

**DOI:** 10.3390/polym14225008

**Published:** 2022-11-18

**Authors:** Hassan Ali Alkadhim, Muhammad Nasir Amin, Waqas Ahmad, Kaffayatullah Khan, Mohammed Najeeb Al-Hashem, Sara Houda, Marc Azab, Zaher Abdel Baki

**Affiliations:** 1 Department of Civil and Environmental Engineering, College of Engineering, King Faisal University, Al-Ahsa 31982, Saudi Arabia; 2 Department of Civil Engineering, COMSATS University Islamabad, Abbottabad 22060, Pakistan; 3 College of Engineering and Technology, American University of the Middle East, Egaila 54200, Kuwait

**Keywords:** fibers, geopolymers, fiber-reinforced geopolymers, bibliographic analysis

## Abstract

This study examined the bibliographic data on fiber-reinforced geopolymers (FRGPs) using scientometrics to determine their important features. Manual review articles are inadequate in their capability to connect various segments of literature in an ordered and systematic manner. Scientific mapping, co-citation, and co-occurrence are the difficult aspects of current research. The Scopus database was utilized to find and obtain the data needed to achieve the study’s aims. The VOSviewer application was employed to assess the literature records from 751 publications, including citation, bibliographic, keyword, and abstract details. Significant publishing outlets, keywords, prolific researchers in terms of citations and articles published, top-cited documents, and locations actively participating in FRGP investigations were identified during the data review. The possible uses of FRGP were also highlighted. The scientometric analysis revealed that the most frequently used keywords in FRGP research are inorganic polymers, geopolymers, reinforcement, geopolymer, and compressive strength. Additionally, 27 authors have published more than 10 articles on FRGP, and 29 articles have received more than 100 citations up to June 2022. Due to the graphical illustration and quantitative contribution of scholars and countries, this study can support scholars in building joint ventures and communicating innovative ideas and practices.

## 1. Introduction

Geopolymer (GP) is a type of inorganic silico-aluminum composite with a 3D network made of a SiO_4_ and AlO_4_ tetrahedral unit structure [1,2,3]. It is manufactured by the interaction of active low-calcium silico-alumina ingredients with alkaline activators [4,5]. Active solid aluminosilicates and activators comprising alkali silicates and metals are required for the production of GPs [6,7,8]. The alkaline solution functions as an activator of alkali, binder, and dispersant [9]. In comparison to cementitious materials, GPs offer the benefits of high initial-age strength, rapid hardening, and a large variety of raw ingredients [10,11]. GPs use less energy and emit fewer pollutants during manufacture, and they are regarded as the material having the greatest potential to substitute cement [12,13,14]. The concept of GPs was proposed in 1978 to explain inorganic aluminosilicate polymers made using natural ingredients [15,16,17]. The intention was to utilize alkali metal silicate solutions to encourage the formation of polymeric aluminum silicate materials from geological minerals under severe alkaline environments [18,19]. Consequently, various solid silicate raw materials such as fly ash, slag, silica fume, and other wastes were utilized to effectively produce GPs [20,21].

Conventional cementitious materials have reduced durability such as resistance to elevated temperatures and deterioration [22,23]. GP composites effectively solve this deficiency [24,25,26]. However, GPs are comparable to ceramics in that their tensile and flexural strengths are inadequate, and they are very susceptible to microcracks [27,28]. By integrating fibers, the brittleness of GPs may be addressed by increasing the toughness of composites [29,30,31]. The addition of fibers to the GP can inhibit the emergence of fractures while simultaneously enhancing its ductility, toughness, and tensile strength [32,33,34]. In recent years, several researchers have investigated the durability of GPs, which focused mostly on their resistance to abrasion, weathering, freeze–thaw, sulfate, water absorption, chloride ions, and various dry and wet impacts [35,36]. The mechanical performance and durability of composites are enhanced by altering the concentration of the alkaline solution, silicon to aluminum ratio, curing conditions, and the addition of fibers [37,38,39]. The inclusion of fibers increases the material's fracture performance and flexural strength and enhances its toughening process [29,40]. GPs with fiber reinforcement are more durable than cementitious composites of the same grade [41].

Currently, natural fibers, inorganic fibers, synthetic fibers, and steel fibers are the most frequent types of fibers utilized in GP composites [42,43,44]. Numerous research studies have been conducted on synthetic-fiber-reinforced GPs, such as polypropylene (PP), polyvinyl alcohol (PVA), polyethylene (PE), etc., but their manufacturing method contaminates the atmosphere and has difficulty satisfying the needs of sustainable development [45]. The majority of natural fibers is cellulose or plant fibers. Natural fibers have low cost, are lightweight, have strong adhesion, have easy production methods, and are biodegradable and are attracting the attention of academics [46].

As scientists continue to study fiber-reinforced geopolymers (FRGPs), because of the growing worries regarding the initiation and development of cracks, there is an issue regarding knowledge limitations that might prevent the establishment of new research and academic relationships. Therefore, it is essential to develop and use a method that enables scholars to acquire important information from the highly trustworthy sources available. This issue could be resolved with the aid of a scientometric process. Therefore, the purpose of this study is to conduct a scientometric evaluation of the literature data on FRGP investigations that has been made accessible up until June 2022. A scientometric analysis is used to carry out a quantitative examination of vast bibliographic records using advanced tools. This is because the many aspects of the literature cannot be appropriately and completely linked in traditional review studies. Complex elements of advanced studies include scientific visualization, co-citations, and co-occurrence [47,48]. Scientometric evaluations highlight the regions actively engaged in a research issue as well as the outlets with the greatest publications, frequently used keywords, and highly cited researchers and papers. To achieve the objectives of the current study, data from 751 pertinent papers were found using the Scopus search engine. This data included abstracts, keywords, citations, and bibliographic details. Additionally, the restrictions related to the applications of FRGP in the building industry were explored, along with possible remedies to these limits. Due to the graphical interpretation and quantitative records of countries and scientists, this study will assist academics in developing collaborative developments and exchanging fresh concepts and techniques.

## 2. Review Strategy

**Figure 1:**
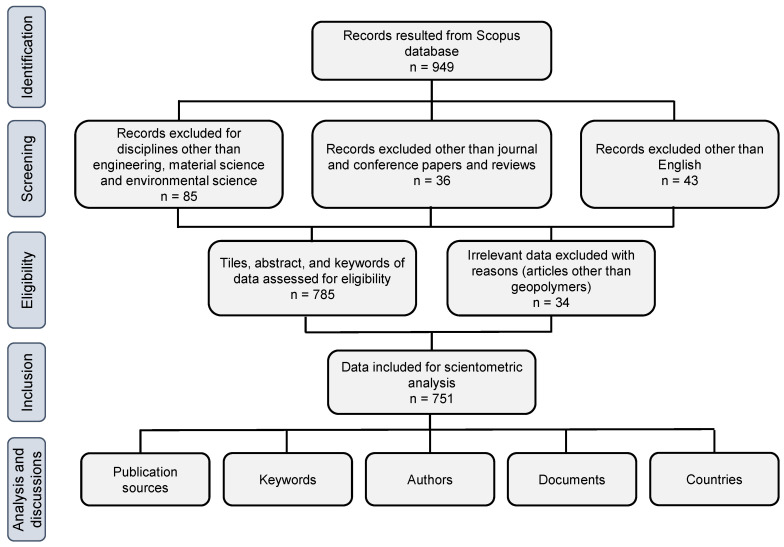
Flowchart of PRISMA technique for data retrieval, filters applied, and analysis.

This work identified the different facets of the literature using a scientometric assessment of bibliometric data. In scientometric studies, systematic visualization, a technique developed by experts for analyzing bibliographic records, is applied [49,50]. Data retrieval was done using the Preferred Reporting Items for Systematic Reviews and Meta-Analyses (PRISMA) approach. The PRISMA checklist is attached as Table S1 in supplementary materials. Since there are extensive articles published on the topic under investigation, it was vital to use a trustworthy database. For this reason, the very dependable databases Scopus and Web of Science were appropriate [51,52]. The Scopus database, which academics strongly suggest [53,54], was used to compile bibliometric data on FRGP research. As of June 2022, 949 results for the term “fiber-reinforced geopolymers” were returned by a Scopus search. There were several filter settings used to reduce unnecessary papers. The whole PRISMA technique for data extraction, assessment, and the various restrictions and filters is shown in Figure 1. A similar strategy was used in some earlier investigations across other subject areas [55,56,57]. Finally, 751 records were used for further analysis using the appropriate tools. The literature data were compiled in Comma Separated Values (CSV) format, and VOSviewer software, version 1.6.18, was used to create the scientific representation and quantitative valuation of the information acquired. VOSviewer is an open-source visualization tool and freely accessible [58,59,60]. Therefore, by using VOSviewer, the intentions of the current study were achieved. The generated data (CSV files) were imported to the VOSviewer to allow for analysis. The systematic research looked at the publication outlets, the highly popular keywords, the highly referenced authors and publications, and countries’ participation. Tables were created to provide quantitative data, while graphs were given to show the different traits, their interactions, and co-occurrence.

## 3. Results and Discussion

### 3.1. Progress on the Research of FRGP

**Figure 2:**
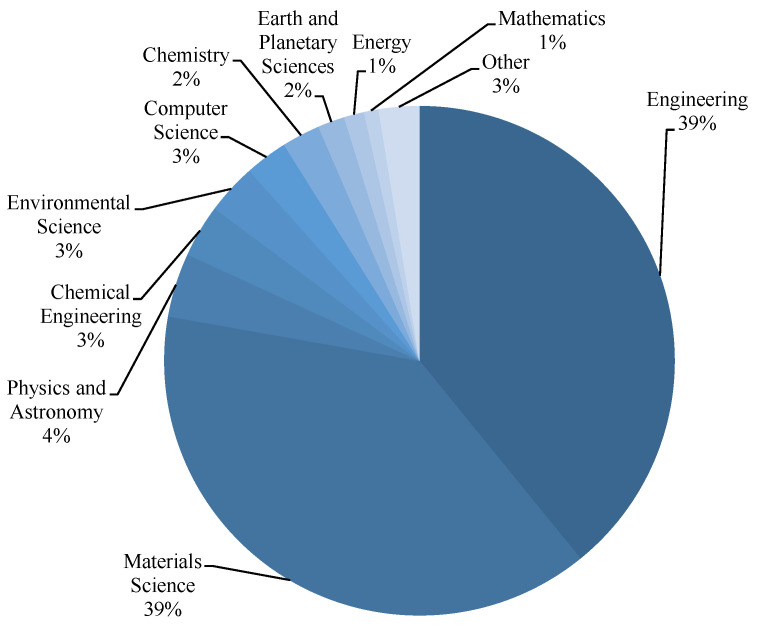
Relevant subject areas containing publications on FRGP studies.

**Figure 3:**
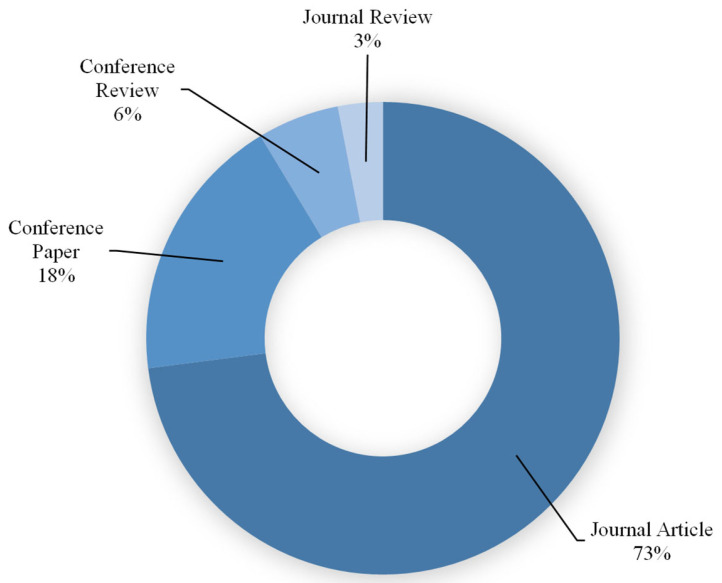
Kinds of documents available on FRGP studies.

**Figure 4:**
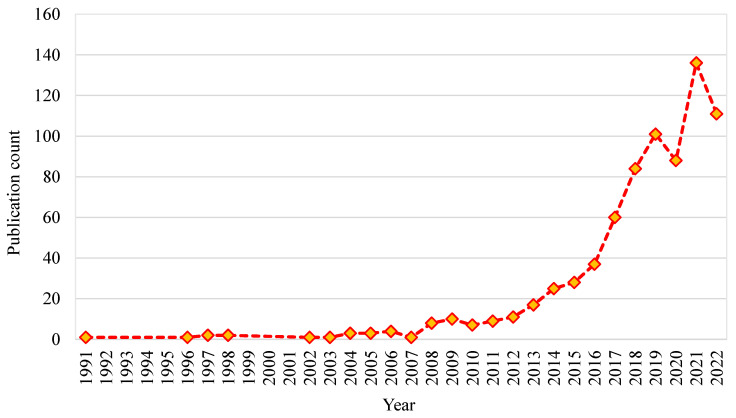
Yearly progress of publications on FRGP studies up to June 2022.

For research development and subject area evaluation, the Scopus analyzer was utilized. As illustrated in Figure 2, engineering and materials science were revealed to be the two top disciplines that produced the most articles, each field producing around 39% of the total papers in the FRGP research. Furthermore, documents related to the study field were searched for on Scopus (Figure 3). According to this study, almost 73%, 18%, 6%, and 3% of all the data comprised journal articles, conference papers, conference reviews, and journal reviews, respectively. Figure 4 shows the annual progress of papers published on FRGP studies from 1991 to June 2022. The initial publication of the FRGP research was discovered to be from 1991. With an average of nearly three articles published yearly up to 2010, there has been a very slight increase in the progress of publications in the area of FRGP investigations. Thereafter, the rate of publications grew gradually, averaging nearly 21 articles per year between 2011 and 2016, with 37 publications in 2015. A significant increase in publications occurred between 2017 and 2021, with a yearly average of about 94 papers and 136 papers in 2021. With 111 articles published so far this year (June 2022), the publications in the field of the subject under study are increasing every year.

### 3.2. Knowledge Visualization of Publishing Outlets

**Figure 5:**
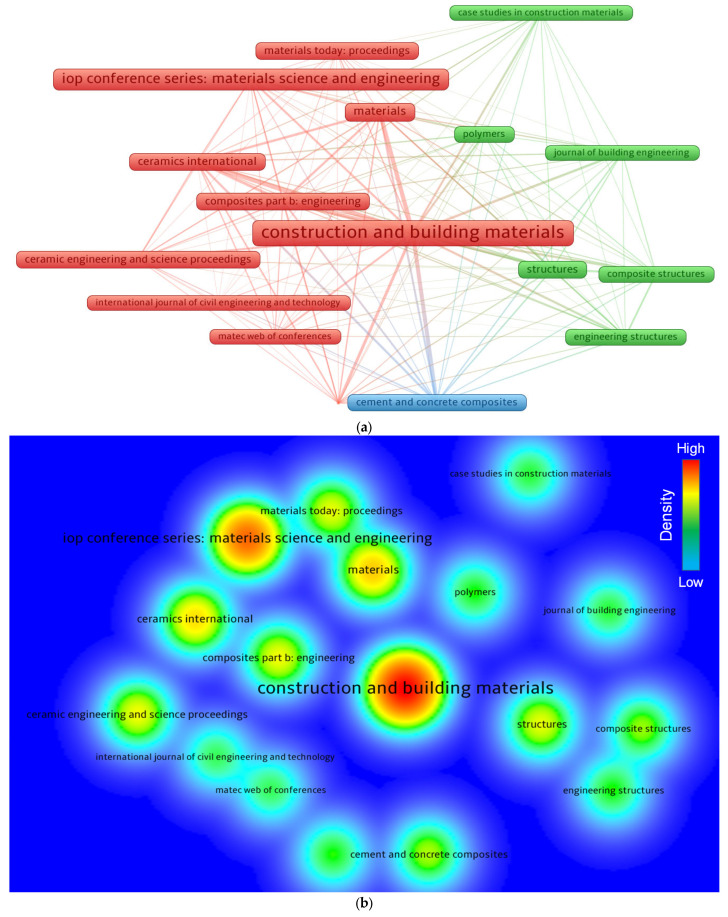
Systematic map of publication outlets: (**a**) network map; (**b**) density map.

Based on bibliographic records, VOSviewer was employed to assess publishing outlets (conferences/journals). A minimum limit of 10 articles was established for an outlet, and 17 of the 206 outlets complied with this requirement. Sources with at least 10 articles on FRGP till June 2022 are included in Table 1, along with the citations obtained during that period. The average citations for each outlet were calculated by dividing the citations with documents. With 95, 57, and 31 documents, respectively, *Construction and Building Materials* (*CBM*), *IOP Conference Series: Materials Science and Engineering*, and *Materials* were found to be the leading three publication outlets in terms of total publications. Moreover, *CBM*, which obtained 3188 citations, *Composites Part B: Engineering*, which obtained 1615 citations, and *Ceramics International*, which received 690 citations, were the top three journals based on citations gained till June 2022. When the comparison of each outlet was made using average citations, the leading outlets were noted to be *Composites Part B: Engineering*, with nearly 77, *Cement and Concrete Composites*, with about 37, and *Journal of Materials in Civil Engineering*, with nearly 35 average citations. In particular, this study would serve as the foundation for the next scientometric analyses of FRGP research. Additionally, past standard review studies were unable to offer a comprehensive summary of the data published. An illustration of sources with at least 10 articles published is shown in Figure 5. The frame size in Figure 5a is connected to the source’s contribution to the topic under investigation based on document count; a greater frame size denotes a stronger influence. For instance, *CBM* has a broader frame than the others, suggesting that it is a key outlet in the subject area. On the map, three clusters were created, each with its unique color (green, red, and blue). Clusters were created using the research outlet’s breadth or how frequently they are mentioned together in relevant articles [61]. The co-citation rates of the sources were categorized by VOSviewer in publications. Ten articles, for instance, were included in the red cluster and were co-cited several times in similar articles. Moreover, in a cluster, linkages between close outlets were stronger than those between widely scattered frames. Compared to *Materials Today: Proceedings* or *MATEC Web of Conferences*, *CBM* showed a greater association with *Composites Part B: Engineering*. Varying colors corresponded to different density concentrations for an outlet, as noticed in Figure 5b. Red, yellow, green, and blue were in order of declining density concentration, with red having the maximum intensity. The red/yellow shades in *CBM*, *Materials*, and other well-known outlets signified a stronger commitment to FRGP investigations. Additionally, the text of some outlets was found to be smaller, faded, and unclear because of the low-density concentration, implying their lower contribution to the research of FRGP.

### 3.3. Knowledge Visualization of Keywords

**Figure 6:**
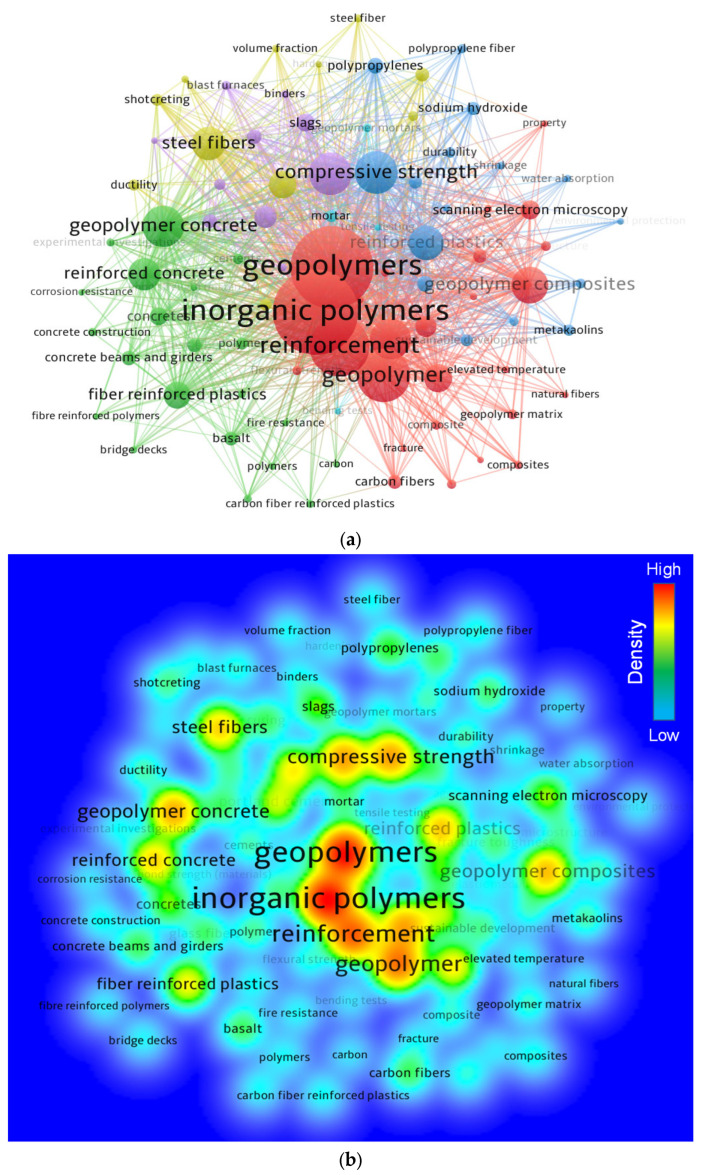
Systematic map of keywords in the research of FRGP: (**a**) visualization map; (**b**) density.

In research, keywords are vital because they differentiate and draw attention to the main domain of the study [62]. The least number of repeats for a term was set at 20, and 88 of the 4261 keywords met this requirement. The top 30 keywords that were used the most frequently in the literature are shown in Table 2. The five very frequent terms in the FRGP research included inorganic polymers, geopolymers, reinforcement, geopolymer, and compressive strength. FRGP has mainly been researched to increase mechanical performance and durability, mainly to decrease brittle behavior by bridging fractures, according to the keyword evaluation. Figure 6 displays a systematic map of terms with relationships, co-occurrences, and densities according to their frequency of occurrence. In Figure 6a, the size of a keyword node reveals its rate of recurrence, and its location reveals where it co-occurs in articles. The leading keywords also have wider nodes on the map than the rest, suggesting that they are important keywords for careful evaluation in the research of FRGP. The graph highlights clusters in a way that shows how frequently they appear together across different publications. The color-coded classification of keywords is based on their co-occurrence in articles. Six clusters of varying colors are shown in Figure 6a. Figure 6b illustrates how different hues correspond to various levels of keyword density. Indicating a higher number of occurrences, inorganic polymers, geopolymers, reinforcement, and other noteworthy keywords are shaded in red/yellow. This discovery will help ambitious scholars choose keywords that will make it simpler to find publications on a specific topic.

### 3.4. Knowledge Visualization of Researchers

**Figure 7:**
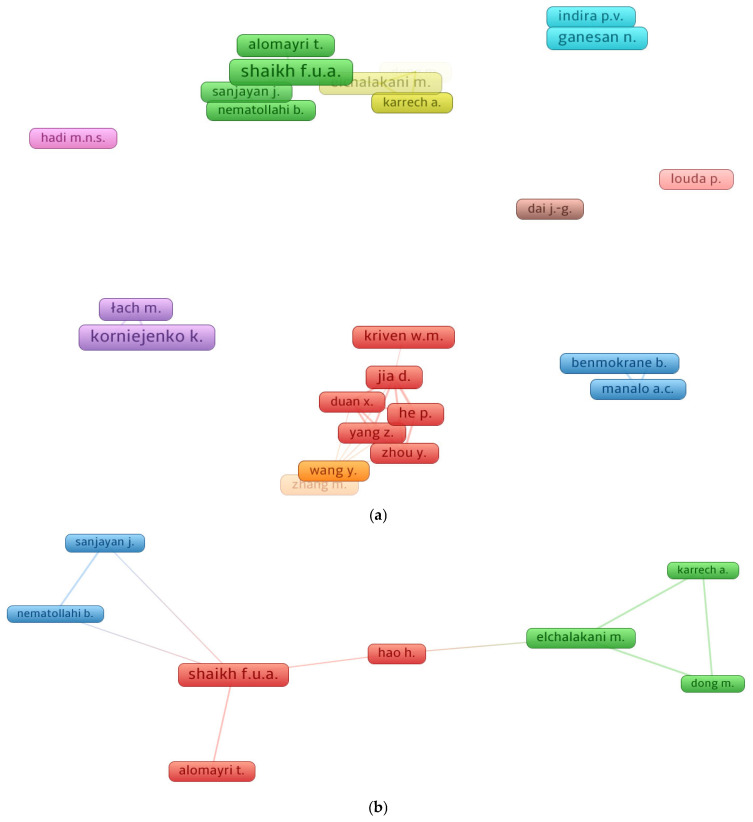
Systematic map indicating author’s collaborations: (**a**) authors with at least 10 articles; (**b**) connected authors based on citations.

Citations serve as evidence of a scholar’s importance in a specific area of research [63]. The minimal publication requirement for a scientist was set at 10, and 27 of the 1583 writers reached this constraint. According to bibliometric data, Table 3 lists the authors of FRGP research with the most publications and citations. By dividing the overall citations by the publications, the average number of citations for a writer was calculated. When all factors, including the quantity of papers, the average citations, and the overall citations, were taken into account, it was difficult to assess a researcher’s success. Instead, each element’s evaluation for the scientist was evaluated separately. Shaikh F.U.A. was found to be the most productive scholar, based on the data analysis, with 26 papers, followed by Korniejenko K., with 25, and Ganesan N., with 18 publications. In the research of FRGP, Shaikh F.U.A. was first in the research area based on total citations with 1486, Alomayri T. was second with 714, and Jia D. was third with 638 total citations. However, Nematollahi B. may be placed at the top with about 62 average citations, Sanjayan J. may be in second place with about 58, and Shaikh F.U.A. may be in third place with approximately 57 average citations. The association between the most well-known writers and authors with at least 10 publications is seen in Figure 7. Figure 7a displays the scientific visualization of the researcher’s co-authorship with at least 10 published articles in the investigation of FRGP. Additionally, Figure 7b shows the greatest number of citation-based related authors. A few FRGP scholars were connected by citations, as it was discovered that 8 of the 27 authors made up the largest group of connected authors based on citations.

### 3.5. Knowledge Visualization of Documents

**Figure 8:**
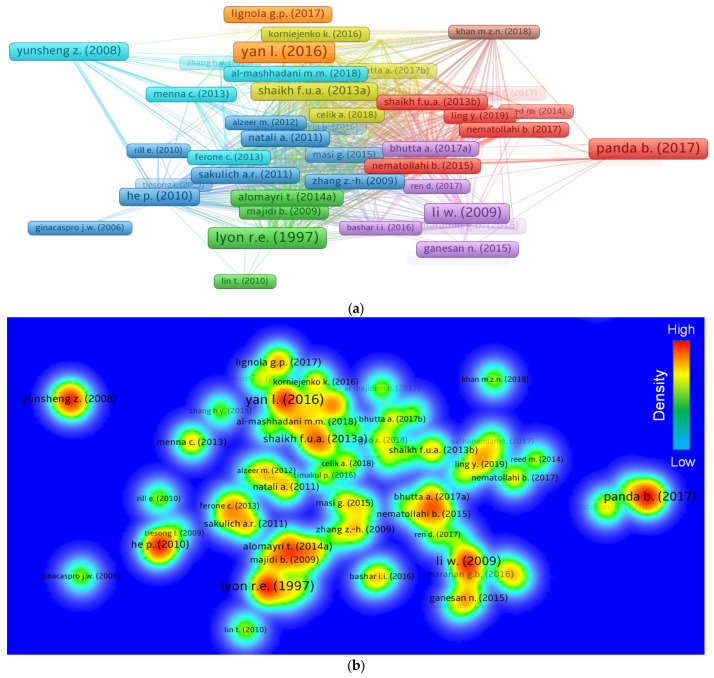
Systematic mapping of papers: (**a**) connected articles based on citations; (**b**) density of connected articles.

An article’s importance in a particular academic field is shown by the number of citations it has obtained. In their respective academic domains, articles having the highest citations are interpreted as revolutionary. A minimum of a 50 citation limit was set for an article, and 84 of 751 articles met this requirement. The top five documents in the field of FRGP, together with their citation details, are shown in Table 4. The article “Geopolymers—Inorganic Polymeric New Materials”, by Davidovits J. [64], had 2553 citations. Additionally, Yan L. [65] and Lyon R.E. [66] were in the top three, with 310 and 291 citations, respectively, for their publications. However, as of June 2022, only 29 papers had received more than 100 citations. The systematic map of papers and their ties to the subject area based on citations is also demonstrated in Figure 8. A map of connected articles with a minimum of 50 citations up to June 2022 is shown in Figure 8a. According to the data analysis, 82 out of 84 articles were linked by citations. As a result, the majority of important papers in the current study field is connected together by citations. The map of density for the linked articles based on citations is shown in Figure 8b. Clearly, papers with more citations showed larger density concentrations.

### 3.6. Knowledge Mapping of Countries

**Figure 9:**
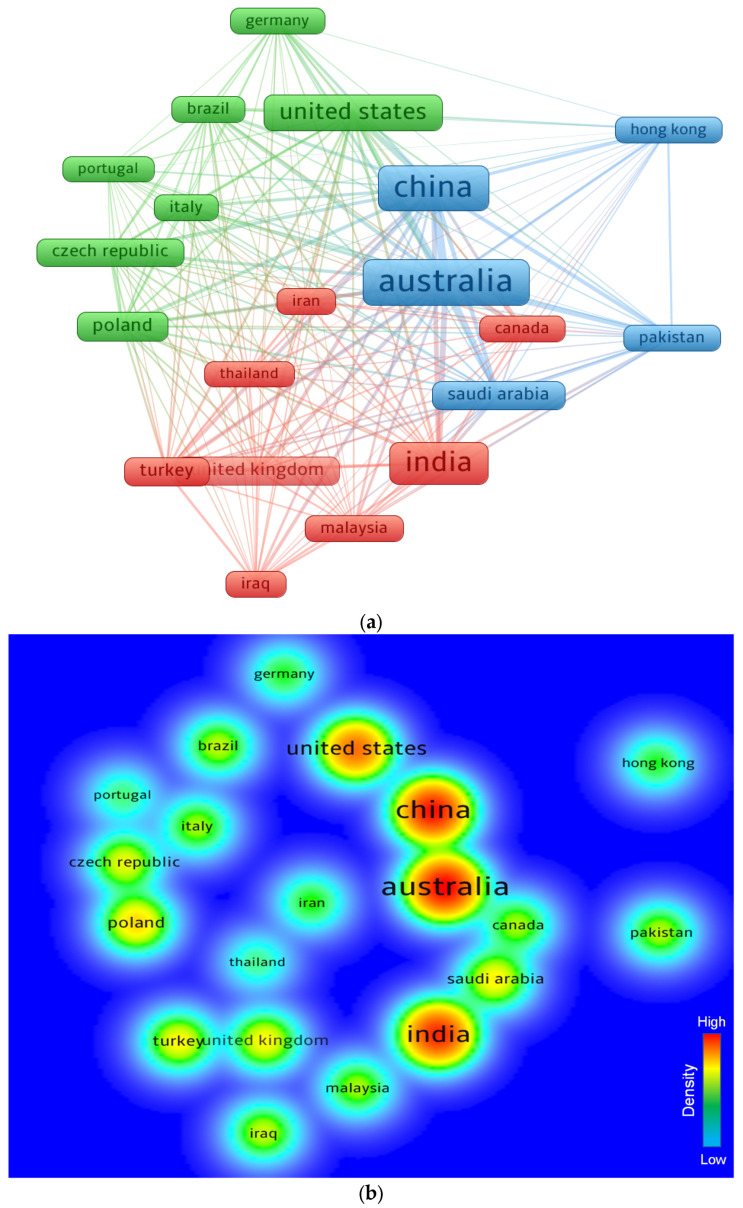
Systematic map of participating countries: (**a**) scientific visualization; (**b**) density.

In comparison to other states, some have contributed more documents to the subject topic and plan to keep on doing so. Readers can view sections of the scientific graph that are specifically for the FRGP study. The least articles limit for a country was set at 10, and 20 countries complied with this condition. According to Table 5, the represented countries have published at least 10 articles on the FRGP study. With 139, 129, and 116 publications each, Australia, China, and India published the most research. The top three countries in terms of citations were also discovered to be Australia (4153 citations), China (3439 citations), and the United States (1864 citations). Figure 9 indicates the scientific framework and the density concentration of the countries connected by citations. According to the quantity of papers released, a country’s influence on a subject is reflected in the frame size shown in Figure 9a. The regions with the top levels of engagement had a larger density concentration, as seen in Figure 9b. Young researchers will be able to develop scientific alliances, start joint businesses, and discuss novel ideas and techniques with the aid of the graphical interpretation and statistical data of the participating states. Researchers from nations with an interest in FRGP investigations can collaborate with experts in the research area and gain from their experience.

## 4. Discussion and Potential Applications

Using literature data, this study carried out a systematic mapping and quantitative assessment of the FRGP research. Past conventional reviews lacked the ability to correctly and completely link various areas of the literature. This research identified the journals and conferences that presented the most articles, the commonly used keywords, the authors and publications that received the most citations, and the countries actively involved in FRGP investigations. The FRGP has mainly been researched to increase mechanical performance and durability, mostly to control brittle behavior by bridging cracks, according to keyword analysis. Additionally, bibliographic data were examined to sort out highly dedicated and prolific writers and countries based on publications and citations. New scientists will benefit from the graphical representation and statistical analysis of active nations and researchers as they establish joint ventures, form scientific alliances, and exchange new approaches and notions. Researchers from different countries motivated to further expand their studies on the application of FRGP can collaborate with specialists in the research field and benefit from their experience. This study explored and discussed the potential applications of FRGP based on an assessment of the literature data and a review of the highly pertinent documents.

**Figure 10:**
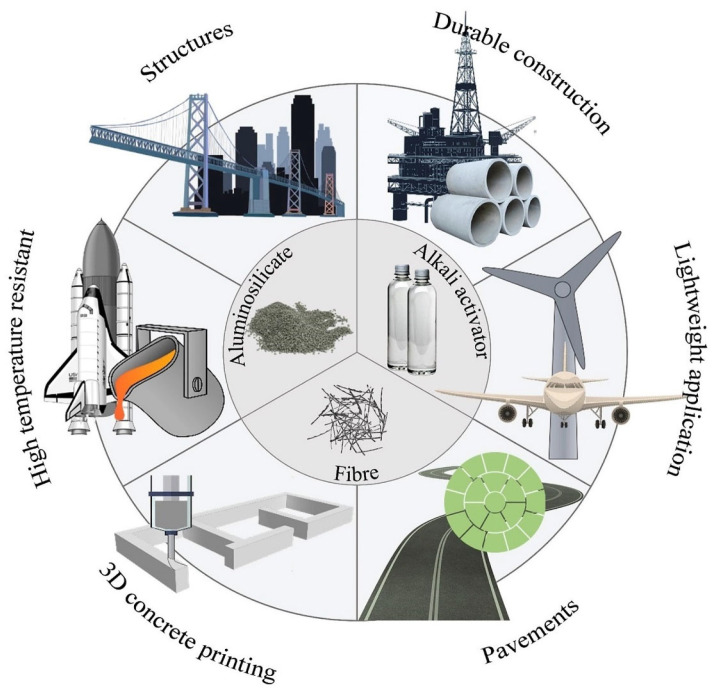
Fiber-reinforced geopolymer’s possible applications [28].

As shown in Figure 10, the application of the material ranged from low-tech/low-cost to high-tech/high-cost, mostly dependent on the type of fiber and binder utilized to form the composites. It is essential to realize that correct conclusions on the effectiveness of each composite should be described separately for various uses. Below are some instances of the uses of FRGP composites.

Due to their accessibility and affordability, steel fibers have been frequently employed in cementitious materials for structural purposes. Steel fibers are incorporated to minimize shrinkage and enhance flexural performance, post-cracking performance, and energy absorption capability of GPs [69]. The highly alkaline nature of GPs maintains the passive condition of the steel reinforcement, hence making it a robust composite for several infrastructure applications [70,71]. Due to the high mechanical strength, flexibility, and hydrophilicity of PE and PVA fibers, niche applications have been examined for PE and PVA-FRGP, such as the progress of strain-hardening GP composites, which require a material with ultra-high impact resistance and ductility [28,72]. A similar use for PP-FRGP has been examined to make the composite more cost-efficient and environmentally friendly [73,74]. In addition, PP and PVA-FRGP have been explored for extruding-based 3D printing processes to construct formwork-less structures with complicated geometries and minimal water curing requirements [67,75,76]. The inflammability of GPs, coupled with the exceptional elevated-temperature resilience of inorganic and carbon fibers, can be utilized in the fabrication of materials where thermal resistance is necessary [66,77]. In addition to their great strength and low weight, carbon fibers have the possibility to be used in the production of lightweight, durable, and robust huge constructions. In addition, carbon-nanotubes were utilized not only to improve the fracture energy of the GPs but also to offer electrical conductivity and piezoresistive responses to examine micro-crack development [78,79]. Inorganic fibers such as silicon carbide and basalt are cost-efficient substitutes for carbon fibers in the production of elevated-temperature GP composites [80,81,82]. Natural fibers are often inexpensive and flexible, and they may be employed as reinforcement in GPs at high concentrations. Several production procedures have been developed and used to address the poor compaction of GPs reinforced with natural fibers. In the presence of 8.3% short cotton fiber, for instance, roller compaction was utilized to drive the GP binder into the fiber system, resulting in a material with a tensile strength of around 32 MPa [83]. However, there is a need for in-depth investigations, methods, and guidelines for large-scale practical applications of FRGP.

## 5. Conclusions

This study conducted a scientometric review of the available literature data on fiber-reinforced geopolymers (FRGP) research to assess several criteria. The Scopus search engine was explored for 751 relevant records and evaluated by employing VOSviewer software. The main findings of this study are as follows:

- The assessment of publication outlets presenting articles on FRGP studies showed that *CBM*, *IOP Conference Series: Materials Science and Engineering*, and *Materials*, are the top publishing outlets based on the number of publications with 95, 57, and 31 documents, respectively. In terms of total citations, the leading three publication outlets are *CBM*, with 3188; *Composites Part B: Engineering*, with 1615; and *Ceramics International*, with 690 citations.
- The assessment of keywords used in the research of FRGP discovered that inorganic polymers, geopolymers, reinforcement, geopolymer, and compressive strength are the five most commonly occurring terms. Additionally, based on the evaluation, it was disclosed that the FRGP has mainly been explored to increase mechanical performance and durability, mainly to reduce brittle behavior by bridging cracks.
- The evaluation of researchers showed that 27 writers had published at least 10 papers up to June 2022. In terms of the number of published documents, overall citations, and average citations, the top authors were considered. Shaikh F.U.A. was determined to be the most prolific author with the most publications (26) and total citations (1486). However, Nematollahi B. was placed at the top based on average citations (almost 62).
- The top countries were analyzed based on their participation in FRGP investigations, and it was found that only 20 countries published at least 10 articles. Australia, China, and India published 139, 129, and 116 articles, respectively. Additionally, Australia obtained 4153 citations, China obtained 3439 citations, and the United States obtained 1864 citations and were positioned as the leading three in terms of citations.
- The potential applications of FRGP composites include elevated temperature resistance, 3D printing, lightweight structures, bridges, and pavements. However, in-depth research, techniques, and guidelines are required for large-scale practical uses of FRGP.

## Figures and Tables

**Table 1:** Most contributing publication outlets in FRGP studies.

S/N	Publication Outlet	Documents	Citations	Average Citations
1	*Construction and Building Materials*	95	3188	34
2	*IOP Conference Series: Materials Science and Engineering*	57	198	3
3	*Materials*	31	498	16
4	*Ceramics International*	27	690	26
5	*Composites Part B: Engineering*	21	1615	77
6	*Ceramic Engineering and Science Proceedings*	21	195	9
7	*Structures*	20	263	13
8	*Materials Today: Proceedings*	20	55	3
9	*Cement and Concrete Composites*	16	598	37
10	*Composite Structures*	15	325	22
11	*Journal of Materials in Civil Engineering*	12	415	35
12	*Engineering Structures*	12	374	31
13	*Polymers*	12	85	7
14	*Journal of Building Engineering*	11	105	10
15	*Case Studies in Construction Materials*	11	41	4
16	*International Journal of Civil Engineering and Technology*	10	32	3
17	*MATEC Web of Conferences*	10	31	3

**Table 2:** List of 30 highly employed keywords in publications of FRGP studies.

S/N	Keyword	Occurrences
1	Inorganic polymers	514
2	Geopolymers	496
3	Reinforcement	326
4	Geopolymer	285
5	Compressive strength	216
6	Fly ash	214
7	Geopolymer concrete	205
8	Fibers	204
9	Geopolymer composites	180
10	Reinforced plastics	167
11	Steel fibers	158
12	Reinforced concrete	152
13	Mechanical properties	127
14	Tensile strength	126
15	Fiber reinforced plastics	120
16	Portland cement	97
17	Bending strength	90
18	Scanning electron microscopy	79
19	Concretes	72
20	Slags	71
21	Polypropylenes	63
22	Fiber reinforced materials	62
23	Concrete beams and girders	55
24	Curing	54
25	Basalt	53
26	Carbon fibers	53
27	Glass fibers	52
28	Fiber-reinforced	51
29	Ordinary Portland cement	51
30	Silicates	50

**Table 3:** Authors together with their publications and citations in FRGP research.

S/N	Researcher	Publication Count	Total Citations	Average Citations
1	Shaikh F.U.A.	26	1486	57
2	Korniejenko K.	25	274	11
3	Ganesan N.	18	220	12
4	Jia D.	17	638	38
5	Hao H.	17	323	19
6	Alomayri T.	16	714	45
7	He P.	16	638	40
8	Kriven W.M.	16	341	21
9	Elchalakani M.	16	186	12
10	Łach M.	15	87	6
11	Indira P.V.	13	69	5
12	Zhou Y.	12	382	32
13	Sanjayan J.	11	634	58
14	Benmokrane B.	11	409	37
15	Manalo A.C.	11	377	34
16	Yang Z.	11	195	18
17	Wang Y.	11	138	13
18	Louda P.	11	126	11
19	Nematollahi B.	10	621	62
20	Maranan G.B.	10	360	36
21	Zhang M.	10	273	27
22	Duan X.	10	193	19
23	Dong M.	10	149	15
24	Karrech A.	10	140	14
25	Hadi M.N.S.	10	97	10
26	Dai J.-G.	10	94	9
27	Mikuła J.	10	90	9

**Table 4:** List of documents having most citations received up to June 2022.

S/N	Article	Title	Citations
1	Davidovits J. [64]	Geopolymers—Inorganic Polymeric New Materials	2553
2	Yan L. [65]	A Review of Recent Research on the use of Cellulosic Fibres, their Fibre Fabric Reinforced Cementitious, Geo-polymer and Polymer Composites in Civil Engineering	310
3	Lyon R.E. [66]	Fire-Resistant Aluminosilicate Composites	291
4	Panda B. [67]	Anisotropic Mechanical Performance of 3D Printed Fiber Reinforced Sustainable Construction Material	265
5	Li W. [68]	Mechanical Properties of Basalt Fiber Reinforced Geopolymeric Concrete under Impact Loading	258

**Table 5:** Detail of regions actively devoted to FRGP investigations.

S/N	Country	Publications	Citations
1	Australia	139	4153
2	China	129	3493
3	India	116	853
4	United States	79	1864
5	Poland	41	530
6	Saudi Arabia	34	699
7	United Kingdom	33	802
8	Turkey	33	557
9	Czech Republic	30	313
10	Iraq	26	331
11	Brazil	25	545
12	Pakistan	24	320
13	Italy	23	820
14	Canada	23	689
15	Malaysia	23	491
16	Iran	18	438
17	Germany	17	599
18	Hong Kong	17	521
19	Portugal	13	396
20	Thailand	12	332

## Data Availability

The data used in this research have been properly cited and reported in the main text.

## Supplementary Materials

The following supporting information can be downloaded at: https://www.mdpi.com/article/10.3390/polym14225008/s1, Table S1. PRISMA checklist.
